# Supplementary material for: A small secreted protein NICOL regulates lumicrine-mediated sperm maturation and male fertility
Source: Nat Commun. 2023 Apr 24;14:2354. doi: 10.1038/s41467-023-37984-x (PMC10125973; doi:10.1038/s41467-023-37984-x)
Supplement: Supplementary file 1 — Supplementary information [file 41467_2023_37984_MOESM1_ESM.pdf]

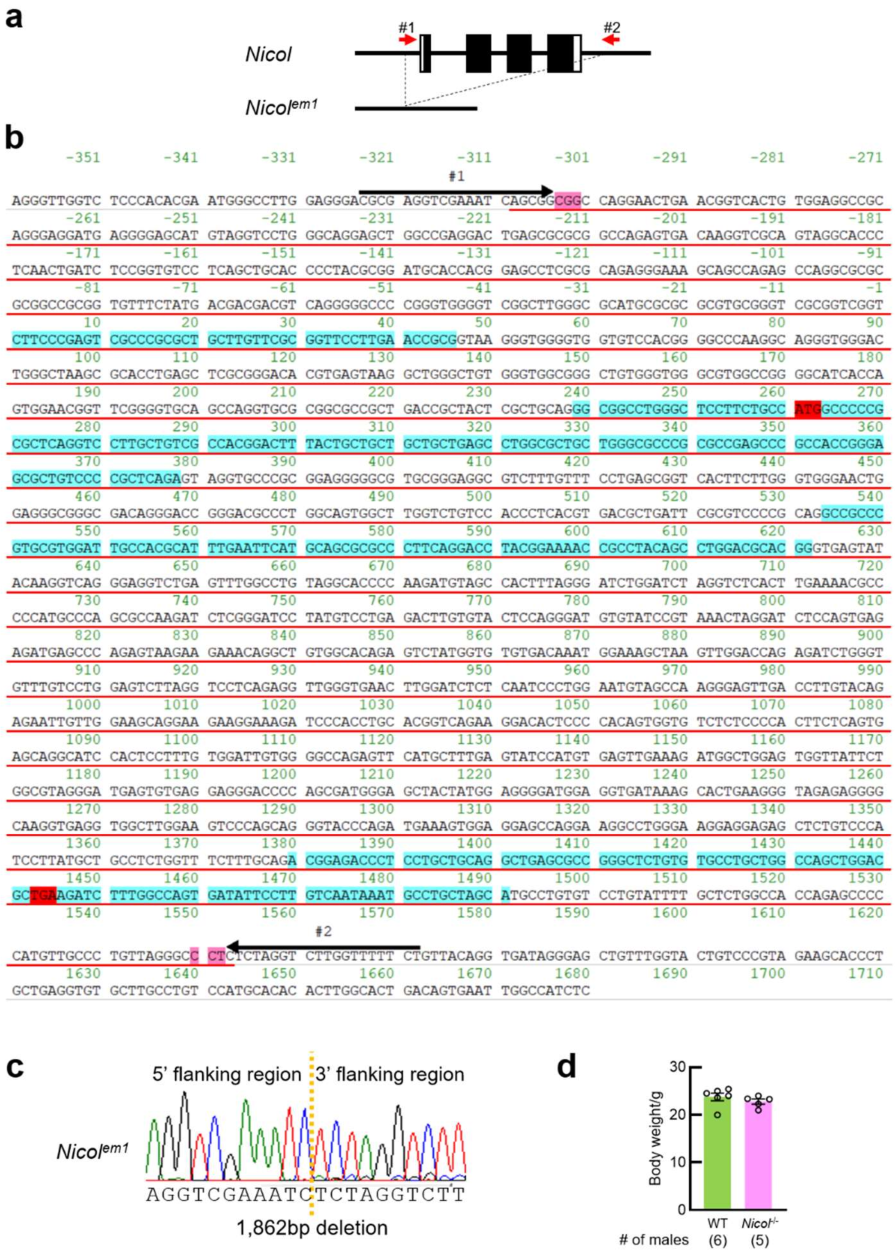

Supplementary Figure 1. Generation of *Nicol* KO mice by CRISPR/CAS9-mediated genome editing. a, *Nicol* gene structure. Boxes represent exons and filled regions indicate protein coding regions. Red arrows indicate crRNA target sites. **b**, *Nicol* targeting strategy. All *Nicol* exons were excised by cleavage at crRNAs #1 and #2 target sites (arrows) after non-homologous end joining repair. **c**, Genomic DNA sequence electropherogram of *Nicol*^{em1}. Non-homologous end joining site indicated by yellow dashed line. **d**, Body weight of 9-week old male mice. Values are shown as mean ± S.E.M. *P* value = 0.3668 (d) was determined by a two-tailed unpaired Students' *t*-test.

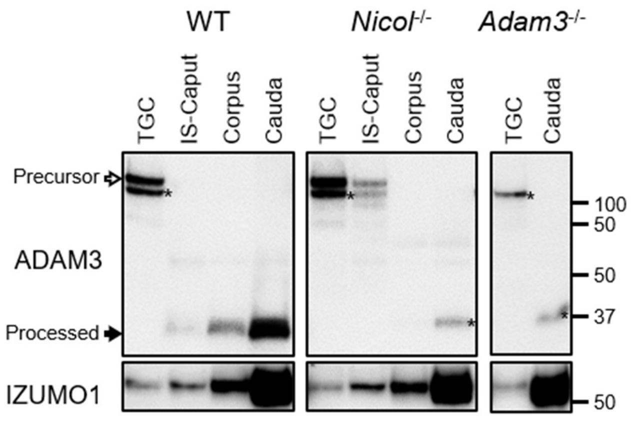

Supplementary Figure 2. ADAM3 processing during sperm transit through epididymis. Immunoblot detection of ADAM3 in TGC; IS-caput, corpus, and cauda epididymal spermatozoa of WT and *Nicol*$^{-/-}$ mice. Asterisks indicate non-specific immunoblot signals which are also present in *Adam3*$^{-/-}$ protein lysates. Images are representative ones obtained from three independent biological replicates.

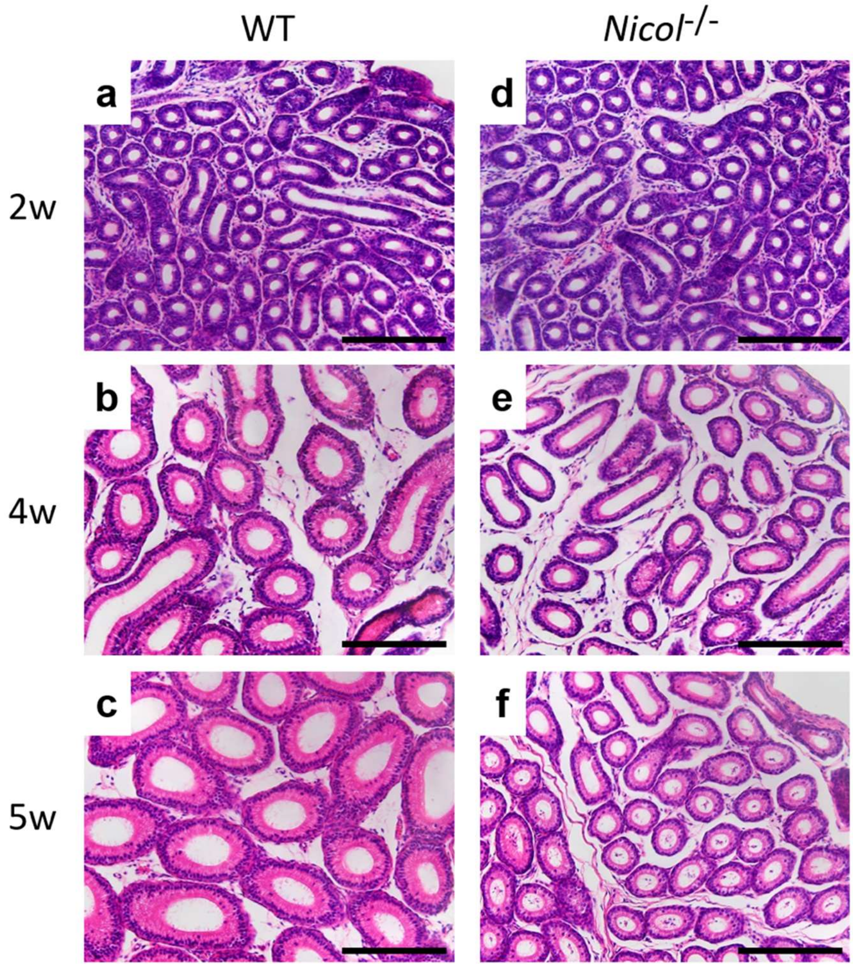

Supplementary Figure 3. Deficient IS differentiation in *Nicol*$^{-/-}$ mice at different ages. a-f, HE staining of IS epididymis in WT mice at 2 (a), 4 (b), and 5 weeks (w), (c) and in *Nicol*$^{-/-}$ mice at 2 (d), 4 (e), and 5 w (f). Scale bars, 200 µm. Images are representative ones obtained from three independent biological replicates.

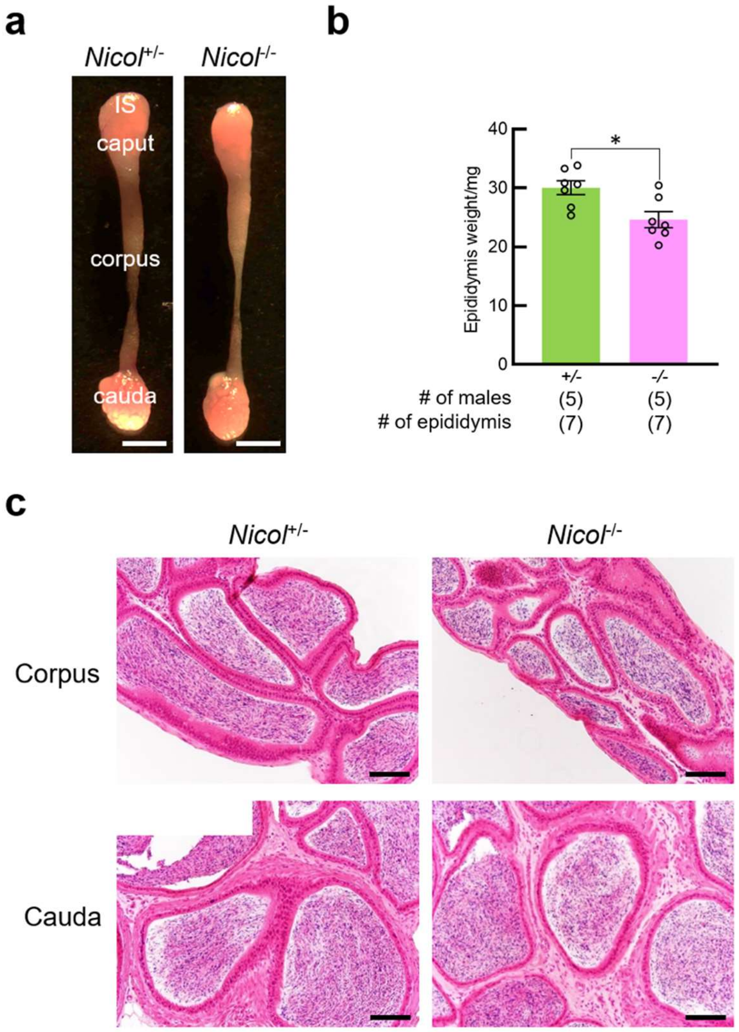

Supplementary Figure 4. Epididymal histology in *Nicol*$^{-/-}$ mice. a, Appearance of *Nicol*$^{+/-}$ and *Nicol*$^{-/-}$ epididymis. Scale bar, 1 mm. **b**, Whole epididymal weights in *Nicol*$^{+/-}$ and *Nicol*$^{-/-}$ male mice. Values are shown as mean ± S.E.M. *P* value = 0.0108 was determined by a two-tailed unpaired Students' *t*-test. **P* < 0.05. **c**, HE staining of *Nicol*$^{+/-}$ (left) and *Nicol*$^{-/-}$ (right) corpus (upper) and cauda (lower) epididymal sections. Scale bar, 100 µm. Images are representative ones obtained from three independent biological replicates (c).

a

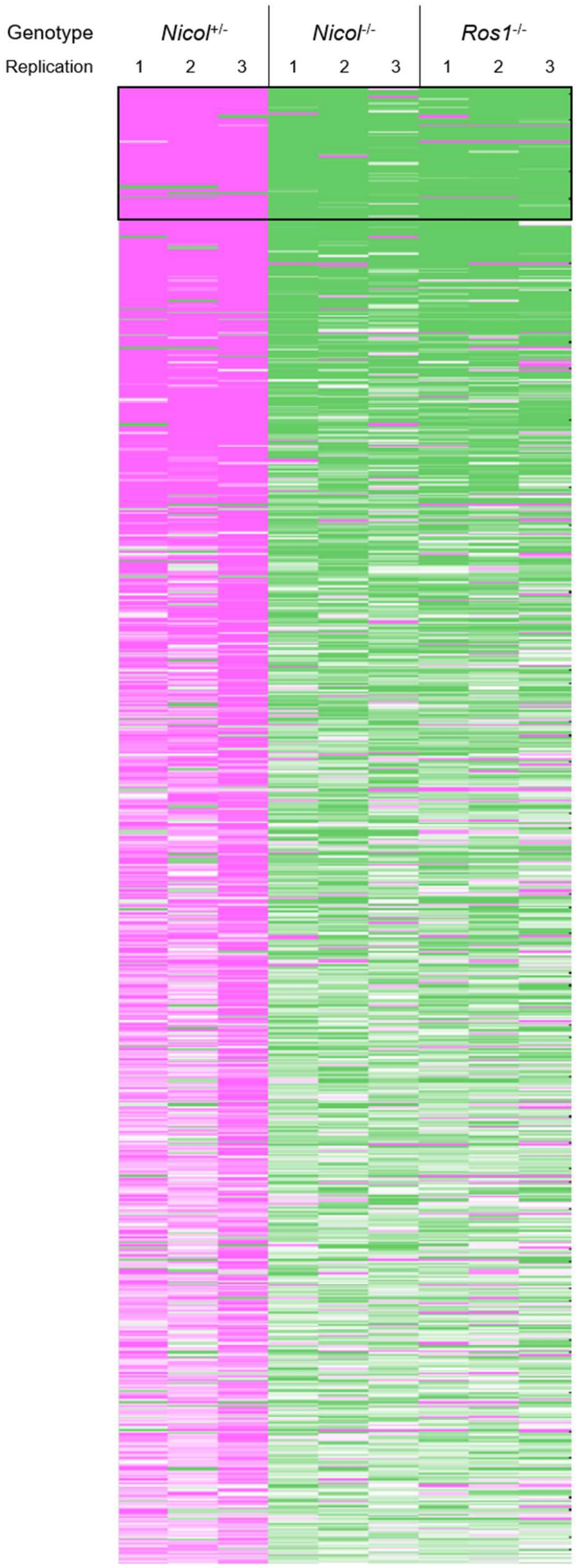

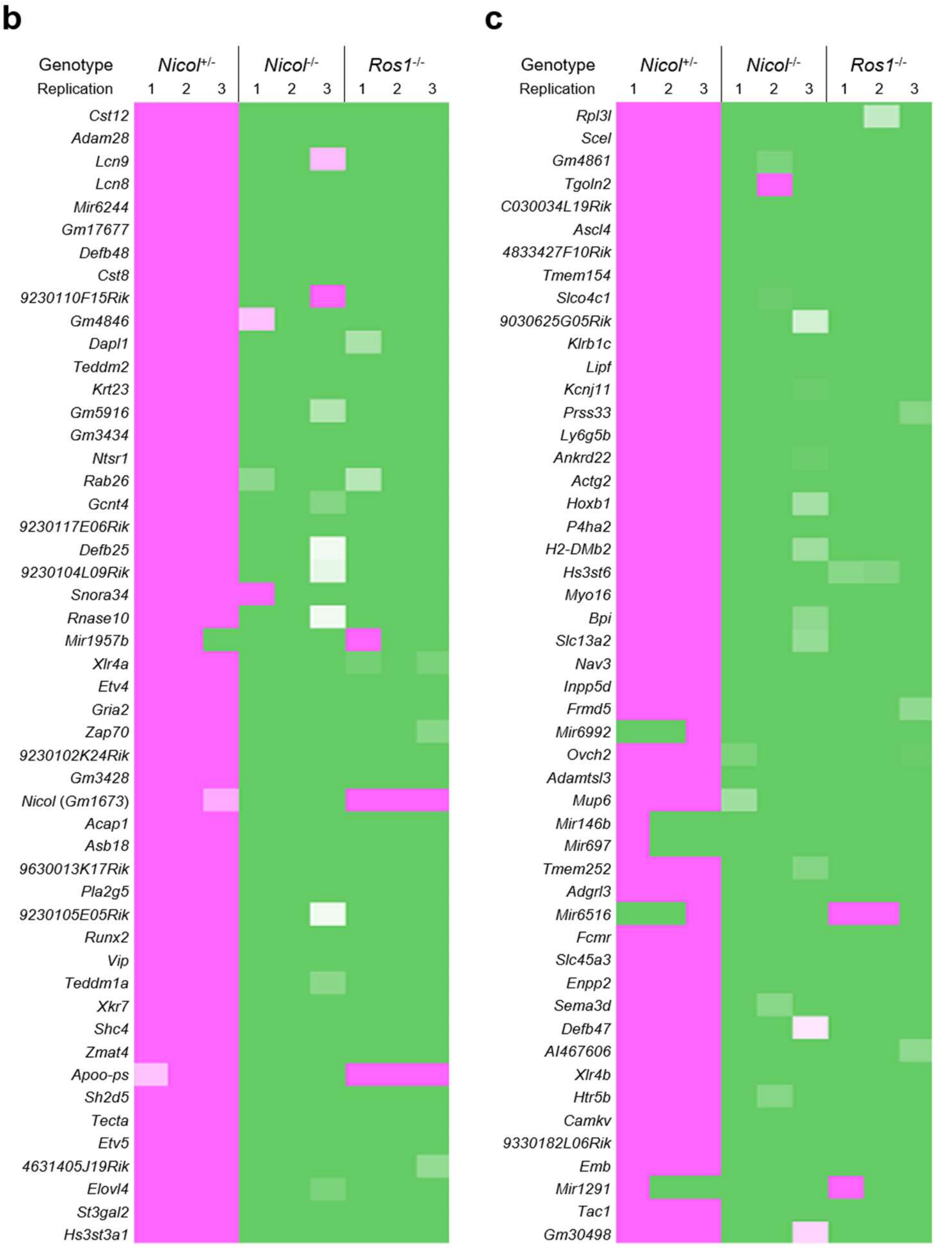

Supplementary Figure 5. Differentially expressed genes between *Nicol*$^{+/-}$, *Nicol*$^{-/-}$, and *Ros1*$^{-/-}$caput epididymis. a, Differentially expressed genes (4,008 genes satisfying $P < 0.05$ determined by a two-tailed unpaired Students' *t*-test) in *Nicol*$^{-/-}$ compared with *Nicol*$^{+/-}$ caput epididymis. *Ros1*$^{-/-}$ IS-caput epididymis shown for comparison. Arbitrary signal intensity acquired from RNA-seq analysis represented by colour (red, higher expression; green, lower expression). **b-c**, Magnified views of top 1–50 (b) and 51–100 (c) genes shown boxed in (a).

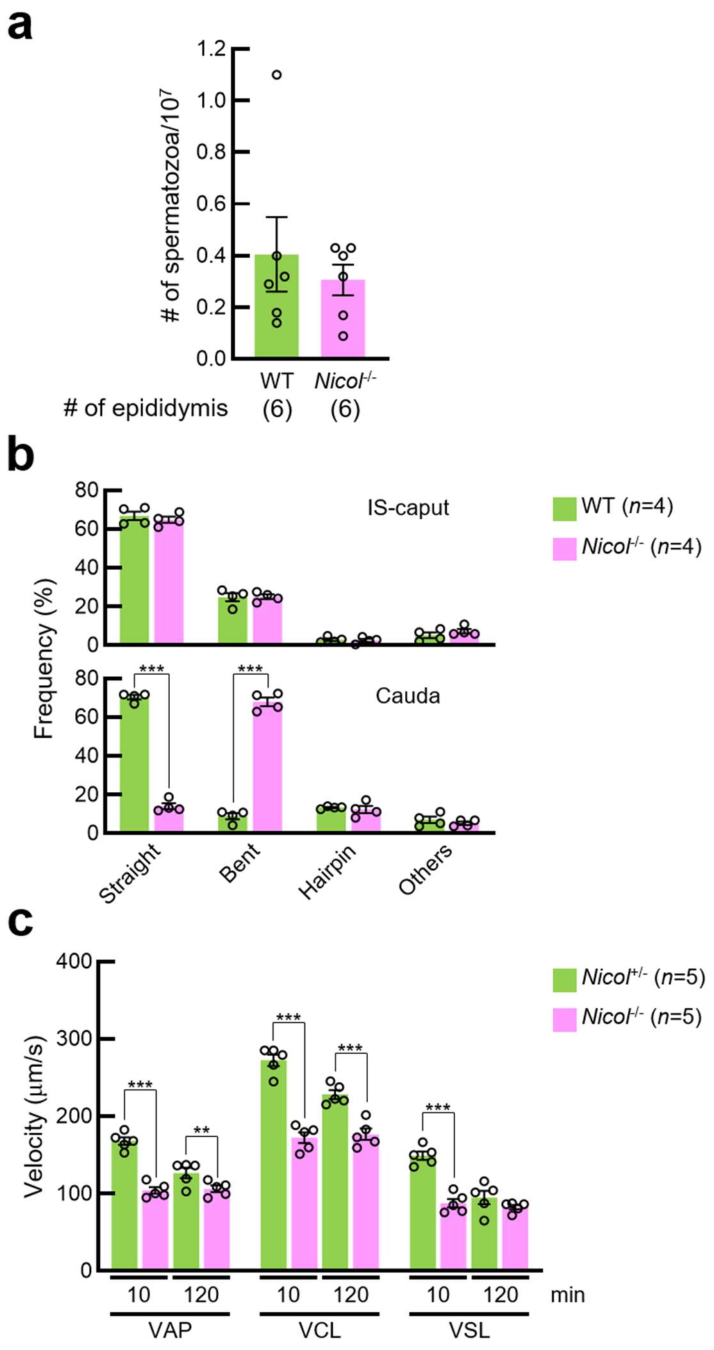

Supplementary Figure 6. Shape and motility of cauda spermatozoa. a, Total number of cauda spermatozoa. **b**, Morphology of spermatozoa isolated from IS-caput and cauda epididymis. **c**, CASA analyses of cauda sperm motility at 10 min and 120 min after suspension. VAP, average path velocity; VSL, straight-line velocity; and VCL, curvilinear velocity. Values are shown as mean ± S.E.M. *P* value = 0.5470 (a), 0.4974, 0.9273, 0.7579, and 0.3096 for straight, bent, hairpin, and others in caput and 0.0000001, 0.0000007, 0.6031,

and 0.3848 for straight, bent, hairpin, and others in cauda (b), 0.00001 and 0.00361 for 10 min and 120 min VAP, 0.00001 and 0.00054 for 10 min and 120 min VCL, and 0.00005 and 0.21883 for 10 min and 120 min VSL (c), respectively, were determined by a two-tailed unpaired Students' *t*-test. *$P < 0.05$, **$P < 0.01$, ***$P < 0.001$.

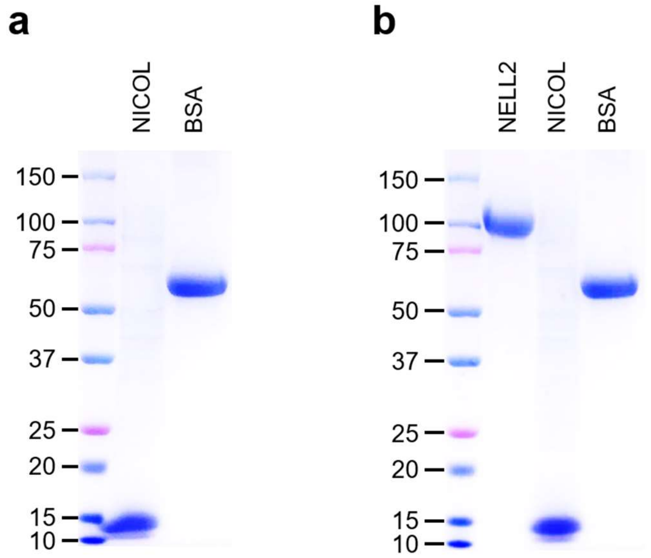

Supplementary Figure 7. Purified NICOL and NELL2 proteins used for pulldown experiment. a-b, Coomassie brilliant blue staining of purified NICOL, purified NELL2, and BSA used for NELL2 pulldown (a) and ROS1 ectodomain pulldown (b) experiments. Images are representative ones obtained from three independent biological replicates (a,b).

Supplementary Table 1. crRNA sequences for CRISPR/CAS9-mediated genome editing.

	Target gene	crRNA sequence
crRNA#1	*Nicol*	5′-CGCGAGGTCGAAATCAGCGG-3′
crRNA#2	*Nicol*	5′-GACCTAGAGAGGGCCCTAAC-3′

Supplementary Table 2. Primer sequences for genotyping gene-modified animals.

Target gene	Primer sequence	Thermal cycling condition	Amplicon size (bp)
Nicol WT	5′-TGGAGACACCGTGACAGGAC-3′ and 5′-ACACCACCCCACCCTTACCG-3′	94°C 15 s<br>65°C 15 s<br>72°C 30 s	626
Nicol KO	5′-TGGAGACACCGTGACAGGAC-3′ and 5′-GACAGGCCAGTATCAAGACC-3′	94°C 15 s<br>65°C 15 s<br>72°C 30 s	441
Clgn-Nicol transgene	5′-TTGAGCGGGCCGCTTGCGCACTGG-3′ and 5′-ATATCGCGTCCAGCTGGCCAGCAGG-3′	94°C 15 s<br>68°C 30 s	543

Supplementary Table 3. Primer sequences for RT-PCR.

Gene	Sequence	Thermal cycling condition	Amplicon size (bp)
Nicol (endogenous)	5′-GCCCGCGCTGCTTGTTCGCGGTTCCTTGAA-3′ and 5′-TGCTAGCAGGCATTTATTGACAAGGAATAT-3′	94°C 10 s<br>60°C 10 s<br>68°C 30 s	374
Nell2	5′-ATGCACGCCATGGAATCCCGGGTGTTACTG-3′ and 5′-TCACAGCTCCTGAAGGCACTGTGGATCCAC-3′	94°C 10 s<br>60°C 10 s<br>68°C 150 s	2,460
Ros1	5′-ATGAAGAACATCTGCTGGCTCACCCTGAAA-3′ and 5′-GGTACCAAGGTGGTACCCACTGATGGTGCT-3′	94°C 10 s<br>60°C 10 s<br>68°C 120 s	2,000
Ptpn6	5′-TCACCGGGACCTCAGCGGGCCTGATGCAGA-3′ and 5′-TGCTCTTGCTATGCACGTTTTCGTACACCT-3′	94°C 10 s<br>60°C 10 s<br>68°C 120 s	1,700
Actb	5′-CATCCGTAAAGACCTCTATGCCAAC-3′ and 5′-ATGGAGCCACCGATCCACA-3′	94°C 10 s<br>60°C 10 s<br>68°C 30 s	171
Nicol transgene	5′-GCTCCTTCTGCCATGGCCCCCGCGCTCAG-3′ and 5′-GTAGGCGGTTTTCCGTAGGT-3′	94°C 10 s<br>60°C 10 s<br>68°C 30 s	204

Supplementary Table 4. Antibody dilutions for immunoblot analysis.

Target protein	Antibody catalogue # or reference	Dilution or final concentration
ADAM2	Millipore, MAB19292	1/1,000
ADAM3	SantaCruz, sc-365288	1/1,000
ADAM28	ProteinTech, 22234-1-AP	1 µg/ml
Calmegin	19	1/1,000
CALR3	20	1/1,000
ERK1/2	Cell Signaling Technology, 4695	1/1,000
Phospho-ERK1/2	Cell Signaling Technology, 4370	1/1,000
ETV5	ProteinTech, 13011-1-AP	1 µg/ml
GAPDH	SantaCruz, sc-25778	1/1,000
Gpx5	SantaCruz, sc-390092	1/1,000
5xHis	Qiagen, 34460	1/1,000
IZUMO1	41	1/1,000
NELL2	ProteinTech, 11268-1-AP	1 µg/ml
OVCH2	10	1 µg/ml
PA	Fujifilm Wako, 012-25863	1 µg/ml
PDILT	21	1/1,000
Rho1D4	Cube Biotech, 40020	1 µg/ml
tACE	22	1/1,000
TOM20	SantaCruz, sc-11415	1/500
Peroxidase-conjugated anti-rabbit IgG	Jackson ImmunoResearch, 111-036-045	1/10,000
Peroxidase-conjugated	Jackson ImmunoResearch, 112-035-167	1/10,000

anti-rat IgG		
Peroxidase-conjugated anti-mouse IgG	Jackson ImmunoResearch, 115-036-062	1/10,000
