# Supplementary material for: A small secreted protein NICOL regulates lumicrine-mediated sperm maturation and male fertility
Source: Nat Commun. 2023 Apr 24;14:2354. doi: 10.1038/s41467-023-37984-x (PMC10125973; doi:10.1038/s41467-023-37984-x)
Supplement: Supplementary file 3 — Reporting Summary [file 41467_2023_37984_MOESM3_ESM.pdf]

nature portfolio

Corresponding author(s): Daiji Kiyozumi, Daniel H Wreschner, Masahito Ikawa

Last updated by author(s): Mar 24, 2023

Reporting Summary

Nature Portfolio wishes to improve the reproducibility of the work that we publish. This form provides structure for consistency and transparency in reporting. For further information on Nature Portfolio policies, see our Editorial Policies and the Editorial Policy Checklist.

Statistics

For all statistical analyses, confirm that the following items are present in the figure legend, table legend, main text, or Methods section.

n/a	Confirmed	
☐	☒	The exact sample size (n) for each experimental group/condition, given as a discrete number and unit of measurement
☐	☒	A statement on whether measurements were taken from distinct samples or whether the same sample was measured repeatedly
☐	☒	The statistical test(s) used AND whether they are one- or two-sided<br>*Only common tests should be described solely by name; describe more complex techniques in the Methods section.*
☒	☐	A description of all covariates tested
☐	☒	A description of any assumptions or corrections, such as tests of normality and adjustment for multiple comparisons
☐	☒	A full description of the statistical parameters including central tendency (e.g. means) or other basic estimates (e.g. regression coefficient) AND variation (e.g. standard deviation) or associated estimates of uncertainty (e.g. confidence intervals)
☐	☒	For null hypothesis testing, the test statistic (e.g. F, t, r) with confidence intervals, effect sizes, degrees of freedom and P value noted<br>*Give P values as exact values whenever suitable.*
☒	☐	For Bayesian analysis, information on the choice of priors and Markov chain Monte Carlo settings
☒	☐	For hierarchical and complex designs, identification of the appropriate level for tests and full reporting of outcomes
☒	☐	Estimates of effect sizes (e.g. Cohen's d, Pearson's r), indicating how they were calculated

Our web collection on statistics for biologists contains articles on many of the points above.

Software and code

Policy information about availability of computer code

Data collection	The obtained sequence reads were mapped onto a mouse reference genome (mm10) using TopHat ver. 2.1.1 (Trapnell, C., Pachter, L. & Salzberg, S. L. TopHat: discovering splice junctions with RNA-Seq. Bioinformatics 25, 1105–11 (2009)). Sperm motility data were collected using the CEROS II (software version 1.5; Hamilton Thorne Biosciences, Beverly, MA, USA) sperm analysis system.
Data analysis	Reads per kilobase of exon per million mapped reads (RPKM) values were calculated for each gene using Cufflinks ver. 2.2.1 (Trapnell, C. et al. Transcript assembly and quantification by RNA-Seq reveals unannotated transcripts and isoform switching during cell differentiation. Nat. Biotechnol. 28, 511–5 (2010)). Single-cell transcriptome data were analysed using Loupe Cell Browser 6.1.0 (10X Genomics). Sperm motility data were analysed using the CEROS II (software version 1.5; Hamilton Thorne Biosciences, Beverly, MA, USA) sperm analysis system. Student’s t-test (two-sided) was performed with Microsoft Excel 2019 (Microsoft). Graph representation was performed with GraphPad Prism9.2.0 (MDF).

For manuscripts utilizing custom algorithms or software that are central to the research but not yet described in published literature, software must be made available to editors and reviewers. We strongly encourage code deposition in a community repository (e.g. GitHub). See the Nature Portfolio guidelines for submitting code & software for further information.

Data

Policy information about availability of data

All manuscripts must include a data availability statement. This statement should provide the following information, where applicable:

- Accession codes, unique identifiers, or web links for publicly available datasets
- A description of any restrictions on data availability
- For clinical datasets or third party data, please ensure that the statement adheres to our policy

The main data supporting the results of this study are available from the publisher's website or corresponding authors. RNA-seq data are available at the NCBI GEO under accession number GSE206174.

Human research participants

Policy information about studies involving human research participants and Sex and Gender in Research.

Reporting on sex and gender	n/a
Population characteristics	n/a
Recruitment	n/a
Ethics oversight	n/a

Note that full information on the approval of the study protocol must also be provided in the manuscript.

Field-specific reporting

Please select the one below that is the best fit for your research. If you are not sure, read the appropriate sections before making your selection.

☒ Life sciences ☐ Behavioural & social sciences ☐ Ecological, evolutionary & environmental sciences

For a reference copy of the document with all sections, see nature.com/documents/nr-reporting-summary-flat.pdf

Life sciences study design

All studies must disclose on these points even when the disclosure is negative.

Sample size	In experiments in which statistical test (two-tailed Student's t-test) was done, the sample size was determined as follows: effect size d = 4, alpha error probability = 0.05, and power = 0.8. From these values the sample size for each experimental group is estimated as 3, therefore the sample sizes are 3≥n. Based on this, the actual sample size was determined by the number of animals available; i.e., three to ten, to confirm biological reproducibility but to reduce animals to be sacrificed unnecessarily. This sample size determination is sufficient to examine statistical significance by Student's t-test in this study and is also endorsed by our previous similar research experiences (for example, Kiyozumi et al., Science. 2020).
Data exclusions	No data were excluded from the analyses.
Replication	At least three replication was employed in each experiments to verify reproducibility.
Randomization	Randomization is not relevant to this study because experiments in this study were designed to compare homozygous gene knockout mice with their control wild-type or heterozygous ones.
Blinding	Blinding was done at the level of data analysis such as statistical test.

Reporting for specific materials, systems and methods

We require information from authors about some types of materials, experimental systems and methods used in many studies. Here, indicate whether each material, system or method listed is relevant to your study. If you are not sure if a list item applies to your research, read the appropriate section before selecting a response.

Materials & experimental systems

n/a	Involved in the study
	☒ Antibodies
	☒ Eukaryotic cell lines
☒	☐ Palaeontology and archaeology
	☒ Animals and other organisms
☒	☐ Clinical data
☒	☐ Dual use research of concern

Methods

n/a	Involved in the study
☒	☐ ChIP-seq
☒	☐ Flow cytometry
☒	☐ MRI-based neuroimaging

Antibodies

Antibodies used

Following antibodies are commercially available: anti-ADAM2 (#MAB19292; Millipore), anti-ERK1/2 (#4695, Cell Signaling Technology), anti-phospho-ERK1/2 (#4370, Cell Signaling Technology), anti-ADAM28 (#22234-1-AP, ProteinTech), anti-NELL2 (#11268-1-AP, ProteinTech), anti-ETV5 (#13011-1-AP, ProteinTech), anti-PRSS37 (#HPA020541, Sigma), anti-ADAM7 (#HPA008879, Sigma), anti-FLAG (#F1804, Sigma), anti-ADAM3 (#sc-365288, SantaCruz), anti-Gpx5 (#sc-390092, SantaCruz), anti-TOM20 (#sc-11415, SantaCruz), anti-5xHis (#34460; Qiagen), anti-PA (#012-25863; Fujifilm Wako, Japan), anti-1D4 (#40020; Cube Biotech), peroxidase-conjugated anti-rabbit IgG (#111-036-045; Jackson ImmunoResearch), peroxidase-conjugated anti-rat IgG (#112-035-167; Jackson ImmunoResearch), peroxidase-conjugated anti-mouse IgG (#115-036-062; Jackson ImmunoResearch). Polyclonal antibodies against CLGN, CALR3, PDILT, and OVCH2, mouse monoclonal antibody against tACE and rat monoclonal antibody against IZUMO1 were generated previously and available from the authors' laboratory.

Validation

Mouse anti-mouse ADAM2 (clone 9D2.2, #MAB19292; Millipore, https://www.emdmillipore.com/US/en/product/Anti-ADAM2-Antibody-clone-9D2.2,MM_NF-MAB19292?bd=1) for immunoblot,
Rabbit anti-mouse ERK1/2 (#4695, Cell Signaling Technology, https://www.cellsignal.com/products/primary-antibodies/p44-42-mapk-erk1-2-137f5-rabbit-mab/4695) for immunoblot,
Rabbit anti-mouse phospho-ERK1/2 (#4370, Cell Signaling Technology, https://www.cellsignal.com/products/primary-antibodies/phospho-p44-42-mapk-erk1-2-thr202-tyr204-d13-14-4e-xp-rabbit-mab/4370) for immunoblot,
Rabbit anti-mouse ADAM28 (#22234-1-AP, ProteinTech, https://www.ptglab.co.jp/products/ADAM28-Antibody-22234-1-AP.htm) for immunoblot,
Rabbit anti-mouse NELL2 (#11268-1-AP, ProteinTech, https://www.ptglab.co.jp/products/NELL2-Antibody-11268-1-AP.htm) for immunoblot,
Rabbit anti-mouse ETV5 (#13011-1-AP, ProteinTech, https://www.ptglab.co.jp/products/ETV5-Antibody-13011-1-AP.htm) for immunoblot,
Rabbit anti-mouse PRSS37 (#HPA020541, Sigma, https://www.sigmaaldrich.com/JP/ja/product/sigma/hpa020541) for immunoblot,
Rabbit anti-mouse ADAM7 (#HPA008879, Sigma, https://www.sigmaaldrich.com/JP/ja/product/sigma/hpa008879) for immunoblot,
Mouse anti-FLAG (clone M2, #F1804, Sigma, https://www.sigmaaldrich.com/JP/ja/product/sigma/f1804) for immunoblot,
Mouse anti-mouse ADAM3 (clone F-4, #sc-365288, SantaCruz, https://www.scbt.com/ja/p/adam3-antibody-f-4) for immunoblot,
Rabbit anti-mouse Gpx5 (#sc-390092, SantaCruz, https://www.scbt.com/p/gpx-5-antibody-d-3?requestFrom=search) for immunoblot,
Rabbit anti-mouse TOM20 (#sc-11415, SantaCruz, https://www.scbt.com/ja/p/tom20-antibody-fl-145) for immunoblot,
Mouse anti-5xHis (clone name unknown, #34460; Qiagen, https://www.qiagen.com/us/products/discovery-and-translational-research/protein-purification/tagged-protein-expression-purification-detection/anti-his-hrp-conjugate-kits) for immunoblot,
Rat anti-PA (clone NZ-1, #012-25863; Fujifilm Wako, Japan, https://labchem-wako.fujifilm.com/us/product/detail/W01W0101-2586.html) for immunoblot,
Mouse anti-1D4 (clone 1D4, #40020; Cube Biotech, https://cube-biotech.com/products/protein-purification-products/rho1d4-tag-affinity-resins-magbeads/rho1d4-antibody-0.2-mg/40020) for immunoblot and immunoprecipitation.
Peroxidase-conjugated anti-rabbit IgG (#111-036-045; Jackson ImmunoResearch, https://www.jacksonimmuno.com/catalog/products/111-036-045) for immunoblot,
Peroxidase-conjugated anti-rat IgG (#112-035-167; Jackson ImmunoResearch, https://www.jacksonimmuno.com/catalog/products/112-035-167) for immunoblot,
Peroxidase-conjugated anti-mouse IgG (#115-036-062; Jackson ImmunoResearch, https://www.jacksonimmuno.com/catalog/products/115-036-062) for immunoblot.
Rabbit anti-mouse CLGN, (Ikawa, M. et al. Dev. Biol. 240, 254–61 (2001)) for immunoblot.
Rabbit anti-mouse CALR3, (Ikawa, M. et al. J. Biol. Chem. 286, 5639–46 (2011)) for immunoblot.
Rabbit anti-mouse PDILT, (Tokuhiro, K., Ikawa, M., Benham, A. M. & Okabe, M. Proc. Natl. Acad. Sci. U. S. A. 109, 3850–5 (2012)) for immunoblot.
Rabbit anti-mouse OVCH2, (Kiyozumi, D. et al. Science. 368, 1132–1135 (2020)) for immunoblot.
Rabbit anti-mouse tACE, (Yamaguchi, R., Yamagata, K., Ikawa, M., Moss, S. B. & Okabe, M. Biol. Reprod. 75, 760–766 (2006)) for immunoblot.
Rat anti-mouse IZUMO1, (clone KS64-125, Inoue, N., Ikawa, M., Isotani, A. & Okabe, M. Nature 434, 234–8 (2005)) for immunoblot.

Eukaryotic cell lines

Policy information about cell lines and Sex and Gender in Research

Cell line source(s)

293-F cells were purchased from Invitrogen.

Authentication

Cells were routinely authenticated by morphological examination using microscopy.

Mycoplasma contamination	We confirmed all the cells used in this study were negative for mycoplasma contamination.
Commonly misidentified lines (See ICLAC register)	No commonly misidentified cell lines were used in this study.

Animals and other research organisms

Policy information about studies involving animals; ARRIVE guidelines recommended for reporting animal research, and Sex and Gender in Research

Laboratory animals	Animals: wild-type mouse lines: ICR, B6D2F1; gene-targeted or mutant mouse lines: Nicol-/-, Nell2-/-, Ros1-/-, Adam3-/-, W/Wv; Transgenic mouse lines: Clgn-Nell2, Clgn-Nicol, CAG-Nicol, RBGS (CAG/Su9-DsRed2, Acr3-EGFP).<br>Ages: eight to twelve-week-old male mice were used as adult mice; Two, four, and five-week-old males were also used to analyze postnatal organ development.<br>Sex: Since fertility tests need male and female animals, mice of both sex were used but the research focuses were on males.<br>Housing conditions: Animals were maintained under 12 hours light (8:00-20:00) and 12 hours dark (20:00-8:00) cycle, ambient temperature of 21±1°C, and 55±10% humidity.
Wild animals	This study did not involve wild animals.
Reporting on sex	Sex of animals used are indicated in each experiment.
Field-collected samples	This study did not involve samples collected from the field.
Ethics oversight	All experiments involving animals were approved by the Institutional Animal Care and Use Committees of Osaka University (Osaka, Japan) (the approval ID: H30-01-1) and were conducted in compliance with the university guidelines and regulations for animal experimentation.

Note that full information on the approval of the study protocol must also be provided in the manuscript.
