# Supplementary figures and images for: A small secreted protein NICOL regulates lumicrine-mediated sperm maturation and male fertility
Source: Nat Commun. 2023 Apr 24;14:2354. doi: 10.1038/s41467-023-37984-x (PMC10125973; doi:10.1038/s41467-023-37984-x)

**b**

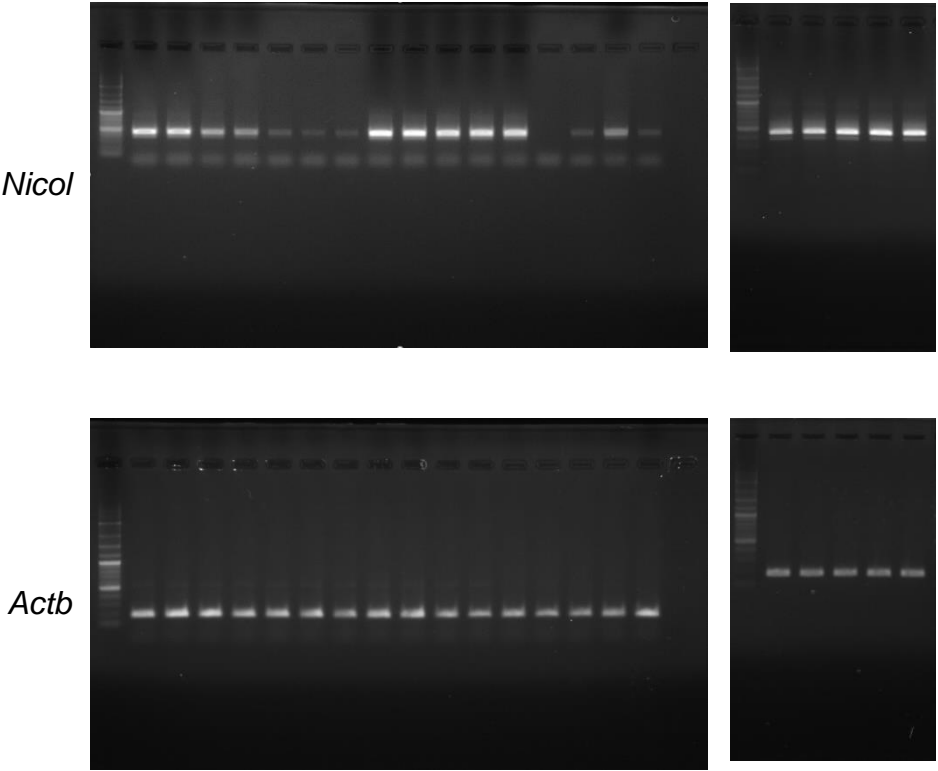

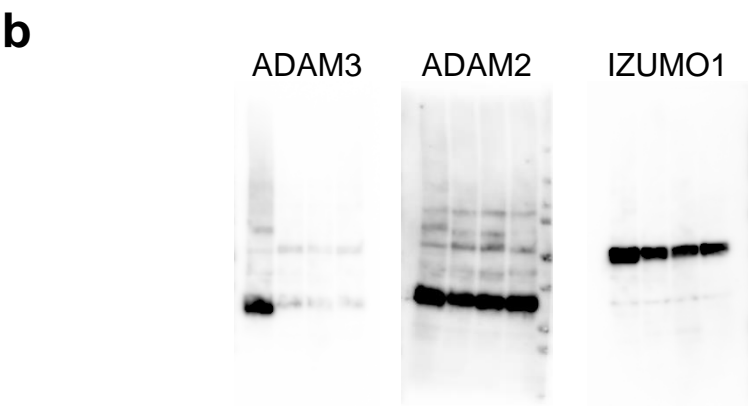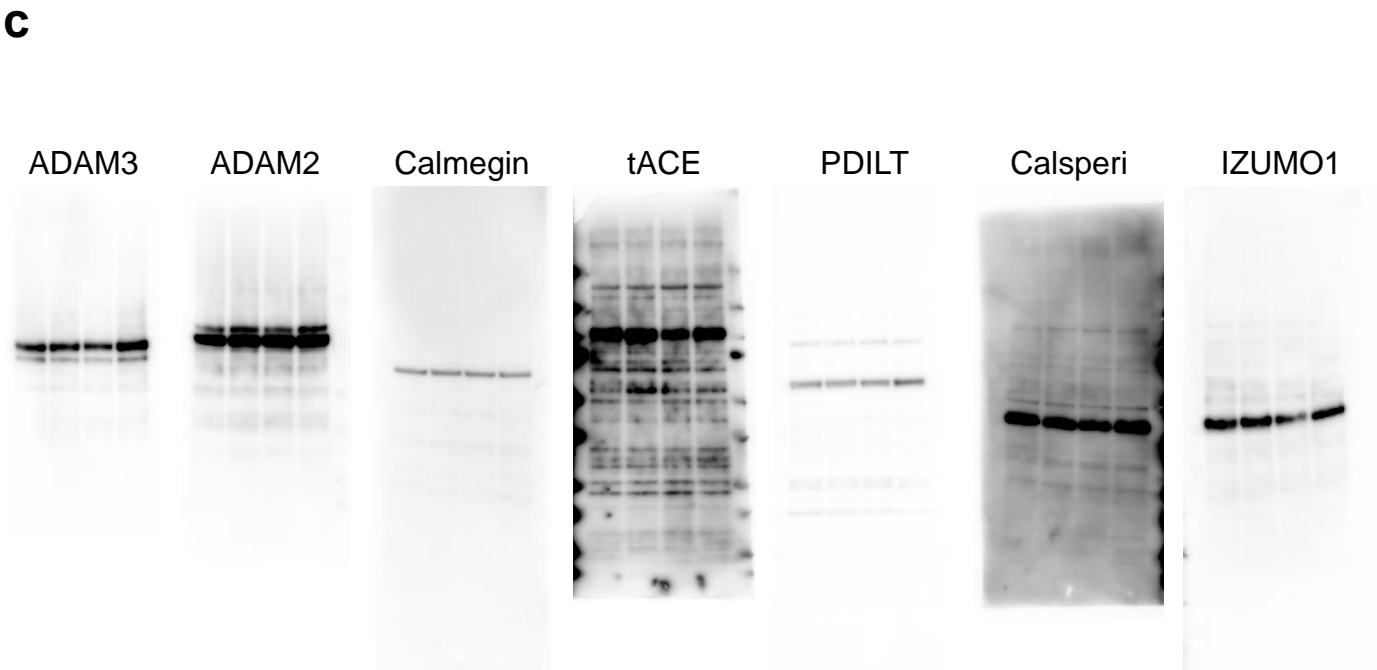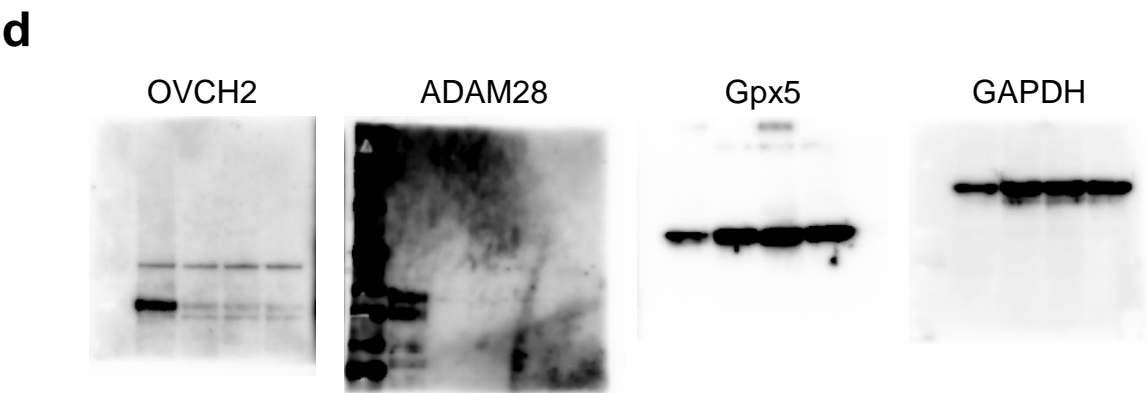

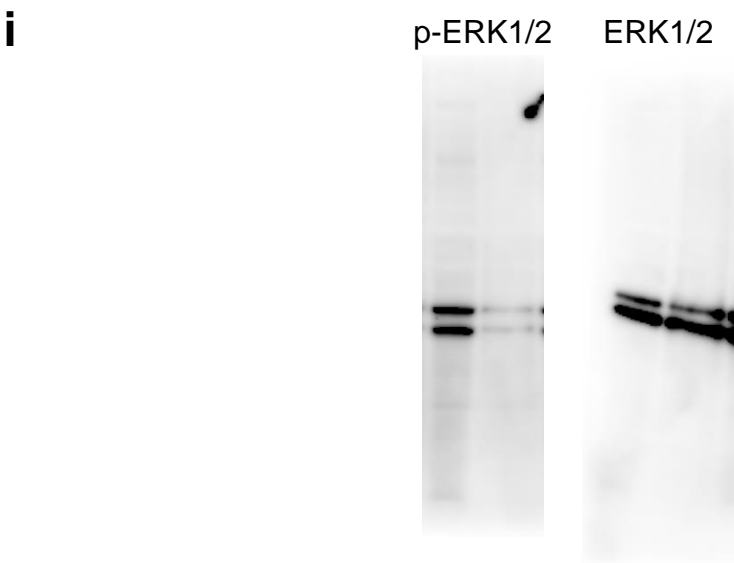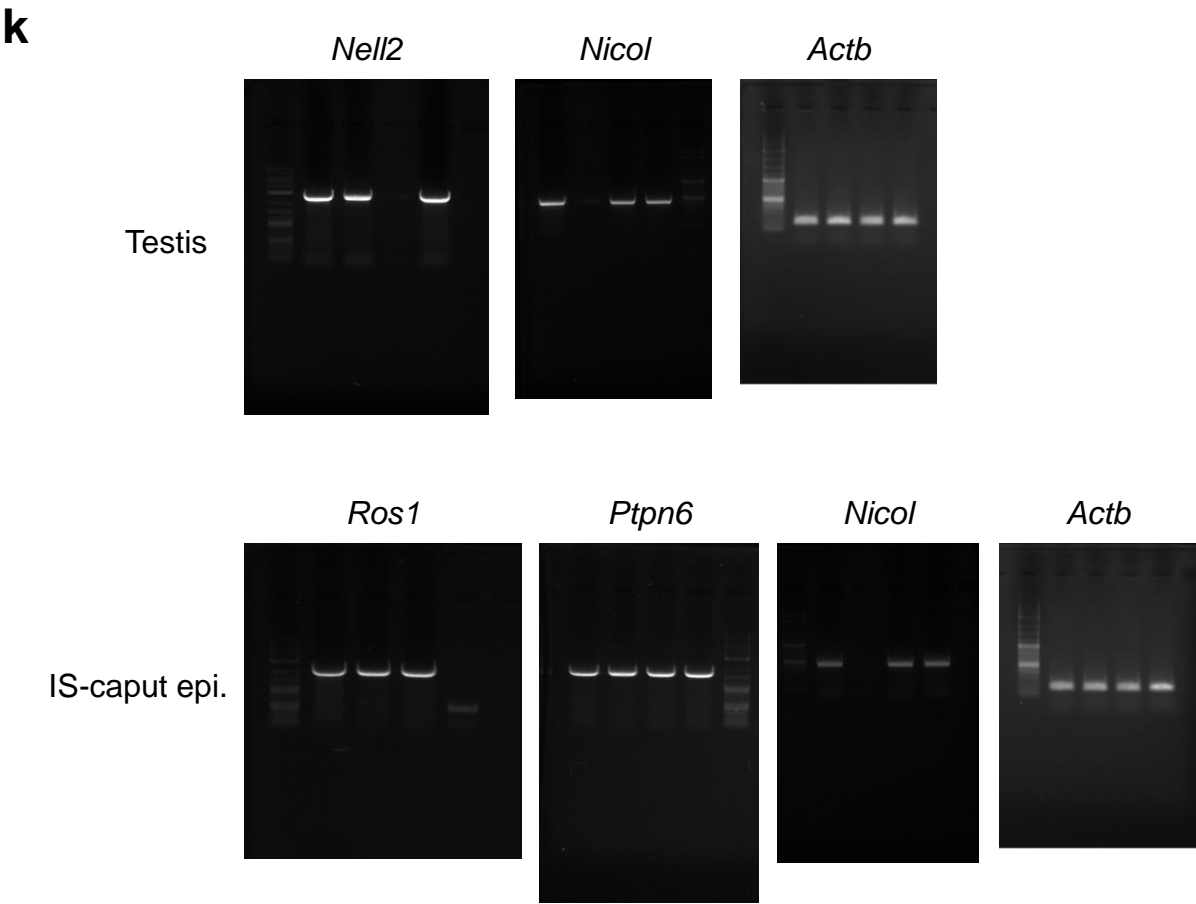

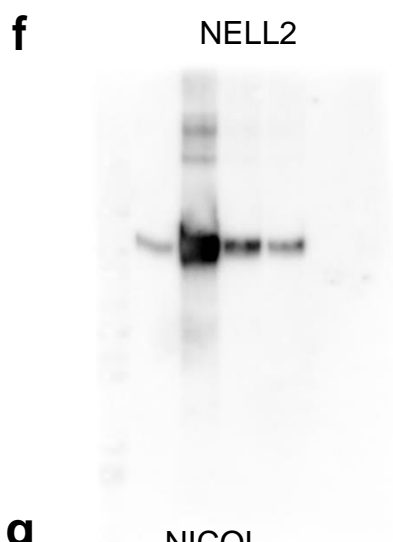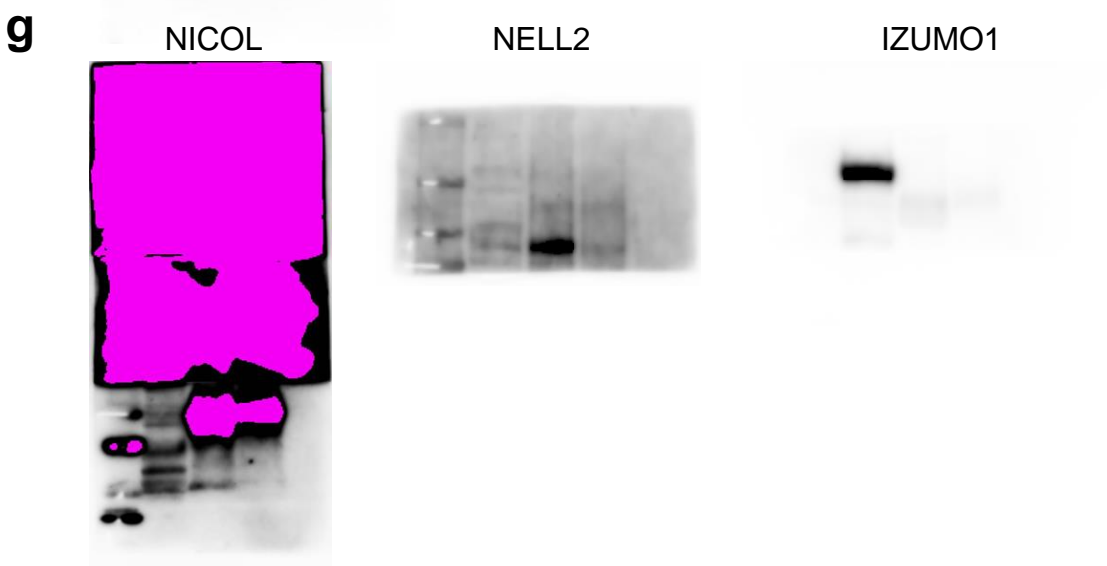

Magenta indicates signal saturation.

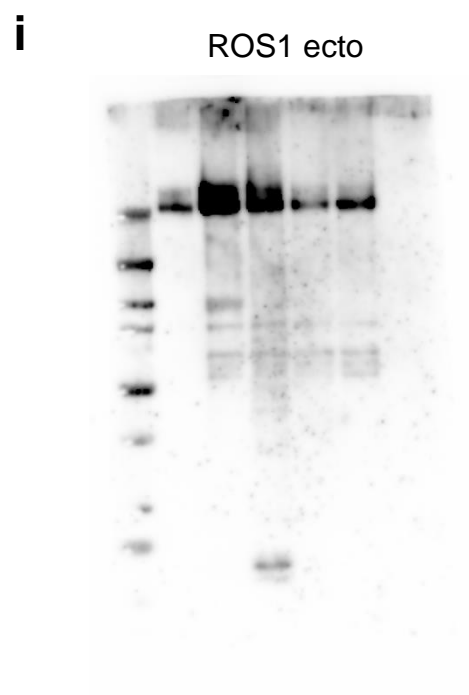

**b**

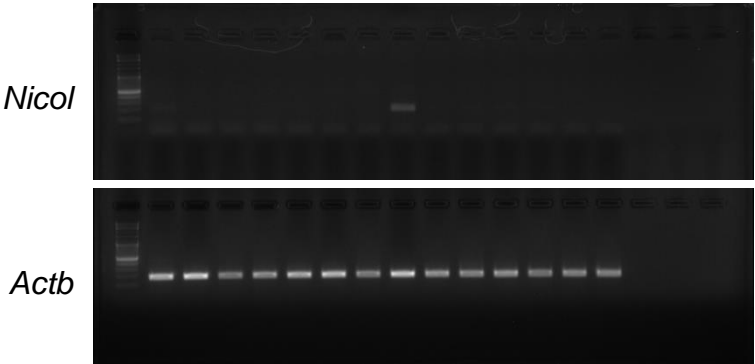

**e**

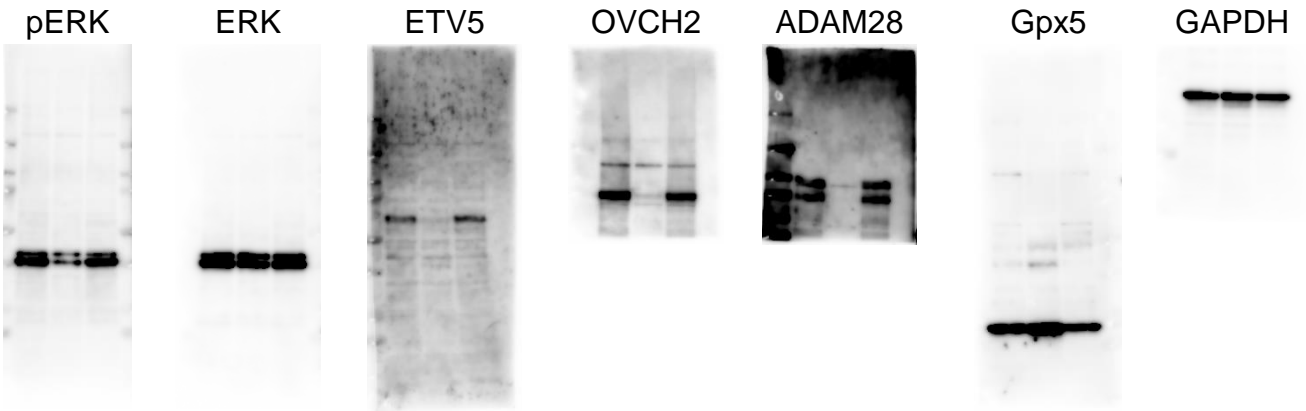

**f**

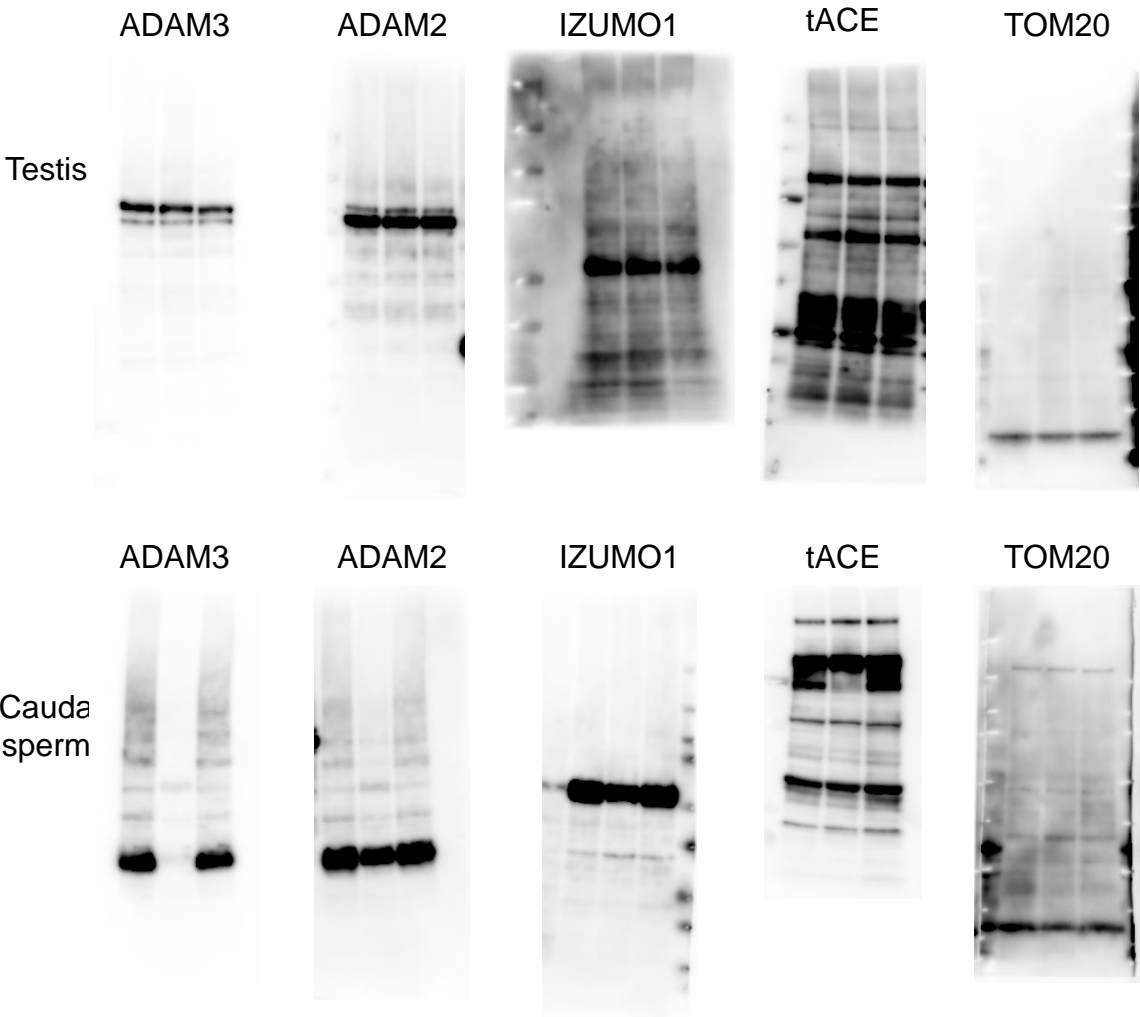

ADAM3

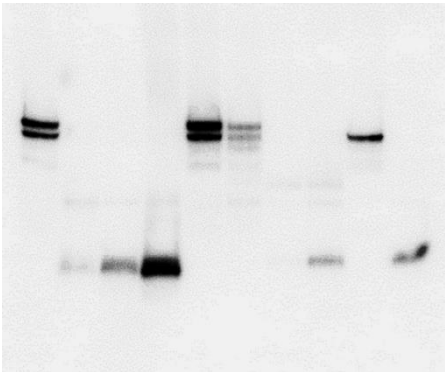

IZUMO1

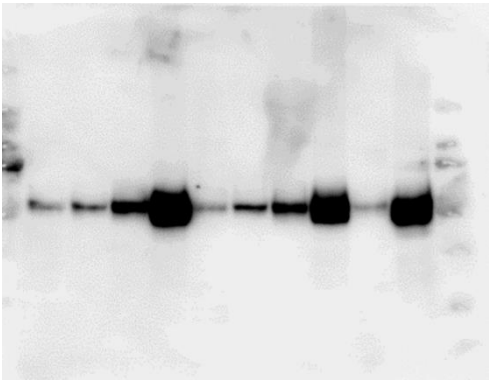

**a**

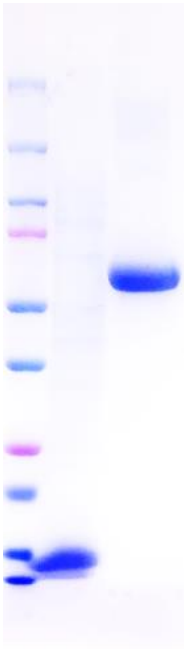

**b**

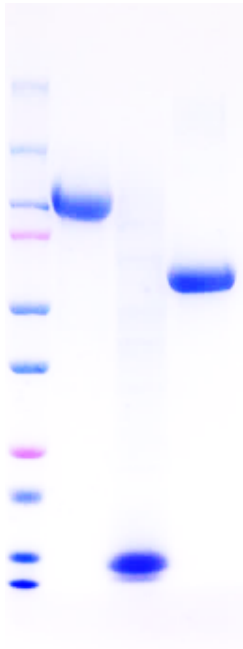

Supplement: Supplementary file 4 — Source data [file 41467_2023_37984_MOESM4_ESM.zip › Source data file and Uncropped gel and blot images file/Uncropped gel and blot images.pdf]
